# New Books

**Published:** 2006-07

Advances in Genetics

Jeffrey Hall, ed.

Burlington, MA:Elsevier, 2006. 224 pp. ISBN: 0-12-017656-4, $149.95

Artists-in-Labs: Processes of Inquiry

Jill Scott, ed.

New York:Springer, 2006. 136 pp. ISBN: 3-211-27957-1, $25.95

Bioinformatics: Genomics and Post-Genomics

Frédéric Dardel, François Képès

Hoboken, NJ:John Wiley & Sons, Inc., 2006. 272 pp. ISBN: 0-470-02001-6, $79.95

Data Mining for Biomedical Applications

Jinyan Li, Qiang Yang, Ah-Hwee Tan, eds.

New York:Springer, 2006. 155 pp. ISBN: 3-540-33104-2, $58

Enhancing Philanthropy’s Support of Biomedical Scientists: Proceedings of a Workshop on Evaluation

George R. Reinhart, ed.

Washington, DC:National Academies Press, 2006. 146 pp. ISBN: 0-309-10097-6, $29.25

Environmental Chemistry at a Glance

Ian Pulford, Hugh Flowers

Malden, MA:Blackwell Publishing, 2006. 144 pp. ISBN: 1-405-13532-8, $29.95

Environmental Impacts of Treated Wood

Timothy G. Townsend, Helena Solo-Gabriele

Boca Raton, FL:CRC Press, 2006. 520 pp. ISBN: 0-8493-6495-7, $139.95

Fundamental Molecular Biology

Lizabeth Allison

Malden, MA:Blackwell Publishing, 2006. 600 pp. ISBN: 1-405-10379-5, $109.95

Geoenvironmental Sustainability

Raymond N. Yong, Catherine N. Mulligan, Masaharu Fukue

Boca Raton, FL:CRC Press, 2006. 400 pp. ISBN: 0-8493-2841-1, $129.95

Human Developmental Toxicants: Aspects of Toxicology and Chemistry

James L. Schardein, Orest T. Macina

Boca Raton, FL:CRC Press, 2006. 472 pp. ISBN: 0-8493-7229-1, $159.95

Human Genetics and Genomics, 3rd ed.

Bruce R. Korf

Malden, MA:Blackwell Publishing, 2006. 488 pp. ISBN: 0-632-04656-2, $54.95

Mercury Hazards to Living Organisms

Ronald Eisler

Boca Raton, FL:CRC Press, 2006. 336 pp. ISBN: 0-8493-9212-8, $169.95

Natural Disasters as Interactive Components of Global-Ecodynamics

Kirill Ya Kondratyev, Vladimir F. Krapivin, Costas A. Varostos

New York:Springer, 2006. 579 pp. ISBN: 3-540-31344-3, $209

Pesticides: Health, Safety and the Environment

G. A. Matthews

Malden, MA:Blackwell Publishing, 2006. 248 pp. ISBN: 1-405-13091-1, $159.99

Principles of Gene Manipulation and Genomics, 7th ed.

Sandy Primrose, Richard Twyman

Malden, MA:Blackwell Publishing, 2006. 668 pp. ISBN: 1-405-13544-1, $94.95

Scaling and Uncertainty Analysis in Ecology

J. Wu, K. B. Jones, H. Li, O. L. Loucks, eds.

New York:Springer, 2006. 313 pp. ISBN: 1-4020-4662-6, $119

The Molecular Biology of Cancer

Stella Pelengaris, ed.

Malden, MA:Blackwell Publishing, 2006. 500 pp. ISBN: 1-405-11814-8, $74.95

The Regulatory Genome: Gene Regulatory Networks In Development And Evolution

Eric Davidson

Burlington, MA:Elsevier, 2006. 304 pp. ISBN: 0-12-088563-8, $69.95

To Recruit and Advance: Women Students and Faculty in U.S. Science and Engineering

Committee on the Guide to Recruiting and Advancing Women Scientists and Engineers in Academia, Committee on Women in Science and Engineering, National Research Council

Washington, DC:National Academies Press, 2006. 150 pp. ISBN: 0-309-10204-9, $33.26

Toxicity Testing for Assessment of Environmental Agents: Interim Report

Committee on Toxicity Testing and Assessment of Environmental Agents, National Research Council

Washington, DC:National Academies Press, 2006. 270 pp. ISBN: 0-309-10092-5, $36

